# Association of Transferrin Gene Polymorphism with Cognitive Deficits and Psychiatric Symptoms in Patients with Chronic Schizophrenia

**DOI:** 10.3390/jcm11216414

**Published:** 2022-10-29

**Authors:** Pinhong Chen, Dongmei Wang, Meihong Xiu, Dachun Chen, Blake Lackey, Hanjing E. Wu, Lubin Wang, Xiangyang Zhang

**Affiliations:** 1 Beijing Institute of Basic Medical Sciences, Beijing 100850, China; 2 CAS Key Laboratory of Mental Health, Institute of Psychology, Beijing 100101, China; 3 Department of Psychology, University of Chinese Academy of Sciences, Beijing 100049, China; 4 Beijing Huilongguan Hospital, Peking University, Beijing 100871, China; 5 Department of Psychiatry and Behavioral Sciences, The University of Texas Health Science Center at Houston, Houston, TX 77030, USA

**Keywords:** transferrin, genetic polymorphism, psychiatric symptoms, cognitive deficits, schizophrenia

## Abstract

A large amount of recent literature has focused on impaired iron homeostasis in the pathophysiology of schizophrenia. Specifically, microarray analysis has illustrated associations between the transferrin locus and schizophrenia. To elaborate on the effects of transferrin on schizophrenia and its psychiatric phenotypes, our study aimed to investigate whether transferrin gene polymorphism was correlated with cognitive deficits and clinical symptoms in schizophrenia. We recruited 564 patients with chronic schizophrenia and 422 healthy controls (HCs) in a Han Chinese population, collected phenotypic data, and genotyped the rs3811655 polymorphism of the transferrin gene. Our results showed that the rs3811655 polymorphism was related to cognitive performance in both patients and HCs, as well as negative symptoms in patients (all *p* < 0.05), and patients carrying at least one G-allele showed worsened cognition/severe negative symptoms (all *p* < 0.05). Further analyses also found that the rs3811655 polymorphism in combination with cognition may exert small but significant contributions to the negative (β = −0.10, t = −2.48, *p* < 0.05) or total psychiatric symptoms (β = −0.08, t = −1.92, *p* < 0.05) in patients. Our findings indicated that the rs3811655 polymorphism may be implicated in the cognitive deficits of schizophrenia and HCs as well as psychiatric symptoms in patients, which suggested the possible iron regulatory mechanism in the pathology of schizophrenia.

## 1. Introduction

Increasing evidence indicates that the dysregulation of iron homeostasis may be involved in the pathophysiology and progression of schizophrenia [1]. Previous studies have demonstrated that serum iron levels and brain iron concentrations in patients with schizophrenia have changed [2,3,4]. In the brain, iron-mediated electron exchange may be involved in a subset of neurobiological processes, such as oxygen transport, neurotransmitter synthesis or metabolism, and the production of myelin [5,6]. The results of a cohort study supported that maternal iron deficiency that was indicated by the reduction of hemoglobin concentration significantly increased the likelihood of schizophrenia spectrum disorders (SSDs) in offspring [7,8,9]. Animal models also demonstrated that severe or moderate iron deficiency induces a risk of developing schizophrenia-like phenotypes [10,11]. For example, in mice, maternal iron deficiency induces early motor abnormalities, prepulse inhibition deficits, imbalances in bioactive amines such as serotonin and dopamine, reduction in hippocampal volume, and impaired cognitive performance [12]. These clinical or experimental kinds of literature have suggested that an imbalance of iron homeostasis may play a pivotal role in the etiology of schizophrenia.

Several studies have shown that impaired iron homeostasis may affect cognitive function in schizophrenia [13,14]. Previous studies have shown that perinatal iron-deficient rats exhibit reduced open-field exploratory behavior and longer path lengths to reach the platform in the Morris water maze test [15]. Pisansky et al. reported that although the impacts of hippocampal iron on prepulse inhibition (PPI) were eliminated, both the hippocampus-specific iron-deficient DMT-1 KO mouse and gestational iron-deficient anemia rat models exhibited impaired PPI in adulthood, which suggested that iron may play a critical role in the development of sensorimotor gating [16]. Several reviews have identified various mechanisms to explain the link between iron deficiency and cognition. For example, it has been observed that iron delimitation may cause tissue hypoxemia, enzyme deficiency, impaired myelin formation, development of oxidative stress, and neurodegenerative processes in the central nervous system, all of which have significant influences on human neurocognitive ability [17]. However, clinical studies reported that patients with low serum ferritin levels exhibit significantly more prominent negative symptoms, but unchanged cognitive performance in first-episode schizophrenia spectrum disorder [18]. Thus, the impacts of iron metabolism on the cognition of schizophrenia still remain speculative.

Another issue to account for is that decreased serum iron levels were found to be associated with psychiatric symptoms in schizophrenia cases such as acute psychotic relapse [19], negative symptoms [20], and catatonia-behavioral syndrome [21]. Moreover, pre-existing cognitive dysfunction in people at clinical high risk for psychosis may predict the transition to psychosis [22]. Notably, real-time declines in cognitive performance may proceed positive symptom expression in schizophrenia [23], and a much-debated question is whether general impairment in verbal fluency, decision making, and executive control might also be related to negative symptoms such as avolition and alogia [24]. This may suggest that the amendment of cognition could promote the recovery of psychotic symptoms [25].

Some psychiatric research has paid special attention to transferrin (TF), because it is the major iron delivery protein in the brain, and iron is critical for neuron survival and myelination [26]. Previous studies have demonstrated decreased serum transferrin levels in treatment-naïve schizophrenia patients [27]; however, some studies have reported normal serum transferrin levels in patients [28,29]. In humans, the gene encoding transferrin is located on chromosome 3q21, encoding a molecule that binds to hemochromatosis (HFE) protein to form a stable complex that regulates iron transport [30]. Furthermore, some researchers believe that transferrin, an iron mediator linked to eicosanoid signaling, is linked to synaptic plasticity and social impairment in people with autism spectrum disorders [31]. Another study discovered that after death, patients with schizophrenia had lower levels of transferrin mRNA expression in the prefrontal cortex of brain [32].

A series of microarray analyses showed that the logarithm of the odds (LOD) scores for the transferrin gene in schizophrenia were between 2.0 and 3.0 [33]. The study also reported that an intronic single-nucleotide polymorphism of the transferrin gene (rs3811655) was strongly associated with schizophrenia, yet there were no significant differences in allele and genotype frequencies of six other SNPs in the transferrin gene between schizophrenia patients and controls [34]. Another study also demonstrated that the investigated HFE mutations (C282Y and H63D) and/or TF-C2 polymorphism were not correlated with schizophrenia/schizoaffective disorder [35]. In our previous study, it had been found that the mediation model of psychiatric symptoms on the relations between Cu/Zn-SOD and cognition in schizophrenia patients may vary with the rs3811655 polymorphism, which may thus act as a modulator in oxidative-stress-induced cognitive dysfunction [36]. Although these studies have been carried out on the transferrin gene and schizophrenia, few studies have examined the correlation pattern of transferrin gene polymorphism on cognitive performance among both schizophrenia patients and community controls, as well as the effects of transferrin gene polymorphism on symptoms of psychosis. Therefore, our study aimed to better understand the association between transferrin gene polymorphism, cognitive deficits, and psychiatric symptoms in a large sample of chronic schizophrenia patients. We hypothesized that (a) whether the association pattern between rs3811655 genotype and cognition among schizophrenia patients would be different from that of healthy controls; (b) whether the rs3811655 genotype was related to psychiatric symptoms in schizophrenia cases; and (c) whether genotype and cognition alone might predict psychiatric symptoms in individuals with chronic schizophrenia.

## 2. Methods

### 2.1. Subjects

A total of 564 patients with chronic schizophrenia were recruited from Beijing Hui-Long-Guan Psychiatric hospital and HeBei Province Veterans Psychiatric Hospital; more than half of the patients (*n* = 319) came from the latter hospital and were all male. Chronic schizophrenia was referred to those with a DSM-IV diagnosis of schizophrenia assessed via the Structured Clinical Interview for DSM-IV (SCID) by two independent psychiatrists and did not meet the criterion of recent-onset schizophrenia as mentioned by Sponheim [37]. Patients were between 25 and 75 years old (average: 47.8 ± 9.0 years) with at least 5 years of disease course (average years: 24.6 ± 8.7 years) and had received long-term antipsychotic treatment for at least 12 months. The minimum effective dose method described by Woods was used to calculate the chlorpromazine equivalent (CPZeq) dose for the first- and second-generation antipsychotic drugs, and patients in our study had a mean CPZeq of 433.6 ± 366.6 mg/day [38].

Healthy controls (*n* = 422; age range 16 to 70 years, average: 46.1 ± 13.2 years) were enlisted from the local community in Beijing during the same recruitment period, and those who have any biological relationship with the patient were not included in the present study. Two research assistants assessed the control subjects’ current mental status and personal or family history of mental disorders to exclude individuals with Axis I disorders.

All patients and healthy controls were Han Chinese. We excluded subjects with medical abnormalities, including central nervous system diseases, neurological disorders, unstable diabetes, hypertension, cardiovascular disease, or drug or alcohol abuse except for tobacco. All subjects voluntarily gave informed consent to participate in this study, which was approved by the Institutional Review Board of Beijing HuiLongGuan Hospital.

### 2.2. Clinical Examination and Cognitive Assessments

A standardized data collection protocol was used to gather demographic and clinical information through patient interviews and available medical records. Four experienced psychiatrists attended a training course to use the Positive and Negative Syndrome Scale (PANSS) during the research preparation stage. After training, repeated assessments showed that the inter-rater correlation coefficient (ICC) of PANSS total score for the four psychiatrists exceeded 0.80.

The translated Chinese version of the Repeatable Battery for the Assessment of Neuropsychological Status (RBANS) [39] was administered to assess neuropsychological function. RBANS consists of 12 subtests that are clustered into a total scale and five index scores, namely, immediate memory, visuospatial/constructional function, language, attention, and delayed memory. The inter-rater correlation coefficient (ICC) of RBANS for 4 experienced psychiatrists was 0.84.

### 2.3. DNA Extraction and SNP Genotyping

DNA was extracted from the whole blood of all participants using the salting-out method [40] and then stored at −80 °C. The genotype of the rs3811655 polymorphism was identified by matrix-assisted laser desorption/ionization time of flight mass spectrometry (MALDI-TOF MS) in the MassARRAY System (Sequenom Inc., San Diego, CA, USA).

### 2.4. Statistical Analysis

One-way ANOVA for continuous variables and chi-squared for categorical variables were used to compare group differences in demographics. The χ^2^ test for goodness of fit was used to compute the Hardy–Weinberg equilibrium in schizophrenia and healthy controls, and χ^2^ tests were used to see if there were any differences in allele and genotype frequencies across groups. After correcting for confounding factors, a logistic regression analysis was used to see if the distribution of the rs3811655 genotype was significantly different between the groups. Using multivariate analysis of covariance (MANCOVA), the effects of genotype on the RBANS total score and its five subscores in patients and healthy controls as well as genotype on PANSS subscale and total scores were studied with gender, age, education, and smoking status as covariates. Non-parametric tests were used to test the main effects of genotype on cognition in the SZ male, SZ female, HC male, and HC female groups, individually. Partial Spearman’s rank correlation analyses were performed to acquire the partial rank correlation between the rs3811655 genotype and cognition/psychiatric symptoms, in which independent variable X and dependent variable Y were regressed out from covariate Z (gender, age, education, and smoking status). After obtaining the probability-scale residuals from the model of X on Z and the model of Y on Z, we then calculated Pearson’s r between the two residuals. Moreover, stepwise multiple regression analysis was employed to investigate the influences of demographic, cognitive, and genotypic parameters on PANSS subscale and total scores. All *p*-values were two-tailed, and the significance threshold was 0.05. The Shapiro–Wilk test, kurtosis, and skewness values were applied to check the normal distribution for all continuous variables. Bonferroni correction was used to adjust for multiple tests, and the G*Power 3.1.9.7 software was employed to calculate the sample sizes for genotypic groups as well as carry out a power calculation. The effect size was calculated as eta-squared and Cohen’s d. The Holm–Bonferroni method [41] was also conducted for multiple correlation correction.

## 3. Results

### 3.1. Clinical Data, and Allele and Genotype Frequencies

The demographic and clinical characteristics of the participants are illustrated in Table S1. The χ^2^ goodness-of-fit test showed that genotype frequencies of the rs3811655 polymorphism were consistent with the Hardy–Weinberg equilibrium in both patients (χ^2^ = 0.26 *p* > 0.05) and controls (χ^2^ = 0.61, *p* > 0.05). Moreover, there were no statistically significant differences in genotype distribution or allele frequency between the two groups (genotype χ^2^ = 0.64, *p* > 0.05; allele χ^2^ = 0.03, *p* > 0.05) (Table S2). A logistic regression analysis was performed to adjust for gender, age, education, and smoking, but still, no significant differences in the genotype distributions and allele frequencies were found (all *p* > 0.05).

### 3.2. Genotypic Effects on Cognitive Functions between Patients and Controls

The total score and subscores of RBANS for patients and HCs are shown in Table 1. MANCOVA omnibus effects are found to be significant for all the cognitive neurometric indices (all *p* < 0.05). We found that diagnosis alone significantly affected the RBANS total and all index scores except for visuospatial/constructional (all *p* < 0.001). There were also significant genotypic effects on RBANS total scores after controlling for covariates (F = 3.12, *p* = 0.045, eta-squared = 0.006), while genotypic effects on language (F = 2.41, *p* = 0.090, eta-squared = 0.005) and attention (F = 2.57, *p* = 0.077, eta-squared = 0.005) index scores approached significance. Marginally significant interaction of genotype × diagnosis was found in delayed memory index scores (F = 2.37, *p* = 0.094, eta-squared = 0.005).

Then, we analyzed the data of patients and controls separately. There were significant genotypic effects on attention (F = 5.55, *p* = 0.004, eta-squared = 0.020) in schizophrenia patients. Bonferroni post hoc analysis revealed that the GG genotype subgroup had a significantly lower attention index score than the CC and GC genotype groups (*p* = 0.003, Cohen’s d = 0.62, 1-β = 0.883; *p* = 0.019, Cohen’s d = 0.55, 1-β = 0.771, respectively). In addition, MANCOVA analysis for HCs showed a significant genotype effect on the delayed memory subscore (F = 3.30, *p* = 0.038, eta-squared = 0.016) and RBANS total score (F = 3.98, *p* = 0.019, eta-squared = 0.019). Post hoc analysis revealed no significant differences in delayed memory and total RBANS scores between genotype groups after Bonferroni correction (all *p* > 0.05).

To further explore sex effects on the association between transferrin gene polymorphism and cognition, we divided the entire sample into four groups: SZ males (*n* = 499), SZ females (*n* = 65), HC males (*n* = 174), and HC females (*n* = 248). As some subgroups had a too small sample size for the GC genotype, non-parametric tests were used for analysis. Kruskal–Wallis H-tests showed that there are significant genotypic effects on the RBANS attention subscore in SZ males (χ^2^(2) = 11.87, *p* < 0.01) as well as the RBANS delayed memory (χ^2^(2) = 1.27, *p* < 0.05) and language (χ^2^(2) = 6.52, *p* < 0.05) subscores in HC females. Pairwise comparisons demonstrated that for SZ males, GG genotype carriers had significantly lower attention subscores than subjects carrying at least one C-allele (all *p* < 0.05), and for HC females, GG genotype carriers showed marginally and significantly lower language and delayed memory subscores than GC carriers (*p* = 0.061, *p* = 0.084, respectively).

### 3.3. Relationship between the rs3811655 Genotypes and Clinical Characteristics in Patients with Schizophrenia

The clinical characteristics of the patients with different genotypes of rs3811655 are summarized in Table 2. MANCOVA omnibus effects were found to be significant for all the psychiatricsymptoms (all *p* < 0.05). Moreover, there was a significant main effect of rs3811655 on PANSS-N score (F = 4.27, *p* = 0.014, eta-squared = 0.015). The Bonferroni post hoc test indicated that compared with subjects homozygous for the C allele, carriers of the GC genotype exhibited more severe negative psychopathology (*p* = 0.048, Cohen’s d = 0.18). The effects of the rs3811655 genotype on psychiatric symptoms were also explored in SZ male and female groups, and a significant genotypic effect on the PANSS-N score (F = 6.66, *p* = 0.001, eta-squared = 0.026) as well as a marginally significant genotypic effect on PANSS total score (F = 2.83, *p* = 0.060, eta-squared = 0.011) in SZ males were noticed, but not in SZ females. The Bonferroni post hoc test revealed that SZ male carriers of GG and GC showed higher PANSS-N scores than the CC genotype subgroup (*p* = 0.010, Cohen’s d = 0.77, 1-β = 0.952; *p* = 0.030, Cohen’s d = 0.24, 1-β = 0.697, respectively). In addition, hemoglobin concentration, as an indicator of iron deficiency, was not significantly different across the genotypic groups in patients, and no effect of genotype on hemoglobin levels was found in SZ male and female subjects (all *p* > 0.05).

### 3.4. Stepwise Multiple Regression Predicting Psychiatric Symptoms from Transferrin Gene Polymorphism, Cognitive Measures, and Demographic Measures among Patients with Schizophrenia

**Figure 1:**
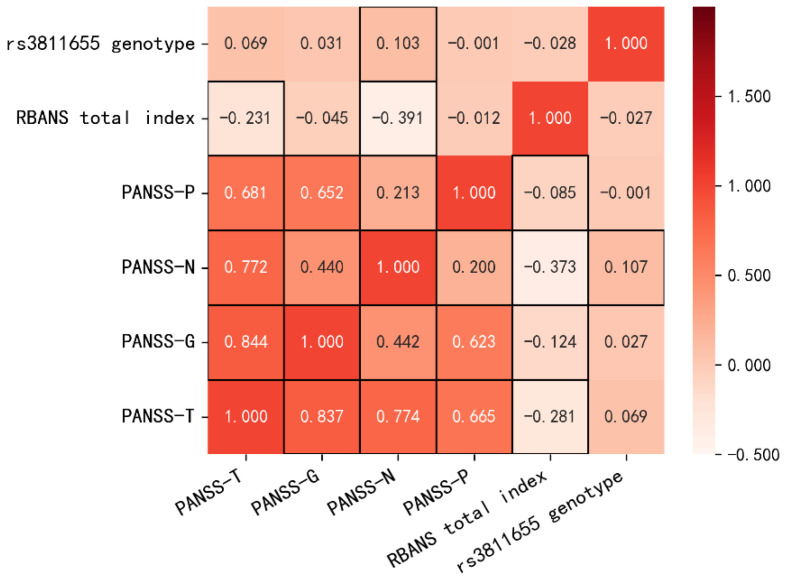
Heat map showing the partial Spearman’s rank correlations between the rs3811655 genotype and cognition/psychiatric symptoms. The upper-left correlations are unadjusted, and the lower-right correlations are partial correlations adjusted for gender, age, education, and smoking status. Shading shows the strength of the correlation with those closer to −1 and 1 being darker. Red boxes are placed around significant correlations.

Figure 1 illustrates pairwise rank correlations between the rs3811655 genotype and cognition/psychiatric symptoms. The unadjusted rank correlations between the RBANS total score and either PANSS-N subscore (ρ = −0.373, *p* = 0.000) or total score (ρ = −0.281, *p* = 0.000) were significantly negative, whereas after adjusting for the covariates, the RBANS total score turned out to be negatively associated with PANSS-P (ρ = −0.085, *p* = 0.044), -N (ρ = −0.373, *p* = 0.000), -G (ρ = −0.124, *p* = 0.003), and -T scores (ρ = −0.281, *p* = 0.000). Moreover, partial Spearman’s rank correlation showed that the rs3811655 polymorphism was significantly correlated with the PANSS negative component (ρ = 0.107, *p* = 0.011), while there were no significant correlations between the rs3811655 genotype and PANSS-P, PANSS-G, and global PANSS scores as well as RBANS subscores and the total score (all *p* > 0.05). However, the significant correlation between the rs3811655 polymorphism and PANSS-N score did not pass the Holm–Bonferroni correction (adjusted *p* = 0.066, adjusted α = 0.008). Furthermore, significant partial Spearman’s rank correlations between the rs3811655 genotype and the PANSS negative component (ρ = 0.142, *p* = 0.002) were found in SZ male patients, but not in SZ females (*p* > 0.05).

Next, a stepwise multiple regression analysis found that RBANS language (β = −0.23, t = −4.93, *p* < 0.001), delayed memory (β = −0.15, t = −2.95, *p* < 0.001) subscales, and rs3811655 GC genotype (homozygous genotype CC as the reference group) (β = −0.10, t =−2.48, *p*< 0.05) were the influencing factors for the PANSS negative symptoms in patients with schizophrenia (Table 3). The model was statistically significant (F (15, 548) = 13.32, *p* < 0.001) and accounted for approximately 24.7% of the variance of PANSS negative symptoms. As listed by the squared semipartial correlations (sr^2^), RBANS language, delayed memory, and transferrin rs3811655 polymorphism uniquely accounted for approximately 4.0%, 2.0%, and 1.0% of the PANSS negative symptoms, respectively. Another stepwise multiple regression analysis revealed that the only predictors of the PANSS total symptoms were daily antipsychotic dose (β = 0.10, t = 2.45, *p* < 0.05, sr^2^ = 1.0%), RBANS language (β = −0.14, t = −2.98, *p* < 0.001, sr^2^ = 2.0%), delayed memory (β = −0.24, t = −4.36, *p* < 0.001, sr^2^ = 3.0%) subscales, and rs3811655 GC genotype (with the CC genotype as the reference group) (β = −0.08, t = −1.92, *p* = 0.055, sr^2^ = 1.0%). F (15, 548) = 8.57, *p* < 0.001, accounted for approximately 16.8 percent of the variance in PANSS total symptoms.

## 4. Discussion

The current study demonstrated three major findings: (a) the rs3811655 genotype was associated with cognitive performance in both schizophrenia patients and community controls, and the association between GG genotype and worsened cognition was only evident among schizophrenia patients or SZ males; (b) negative symptoms varied with rs3811655 genotypes, and patients carrying at least one G-allele showed more severe negative symptoms; (c) the transferrin rs3811655 polymorphism exerted a small but significant contribution to the psychopathology of psychiatric symptoms of schizophrenia.

Our results indicated that the transferrin gene polymorphism (rs3811655) was not associated with the development of schizophrenia. This finding is consistent with other studies that established that a series of transferrin genes polymorphisms, such as rs8177191, rs1799852, rs3811647, TF C2, and TF B variants, are not high-risk genetic variants for schizophrenia in Japanese, Croatian, or Barcelona populations [35,42,43]. However, it was reported that rs3811655 had a strong association with schizophrenia in a Han Chinese population [34]. In addition, microarray analysis of postmortem brain tissue in schizophrenia patients illustrated that myelin-related genes, such as transferrin and myelin-associated glycoprotein, are not associated with myelin abnormalities in schizophrenia [32]. This difference between these association studies may be due to ethnic differences, clinical heterogeneity of schizophrenia, and locus or allele heterogeneity.

Additionally, our study observed that the rs3811655 genotype was associated with cognitive performance in either schizophrenia patients or community controls, both with a small effect size. This indicated that either patients or controls with different genotypes of rs3811655 exhibited subtly unequal cognition. Some important findings came from a twin MRI study in healthy adults, which showed that additive genetic determinants of serum transferrin levels and brain microstructure were partially overlapped. The hemochromatosis HFE gene polymorphism (H63D at rs1799945) is also associated with reduced transferrin levels and white matter fiber integrity in the external capsule [44]. Another study showed that the abnormal homeostasis of iron may inhibit myelination and reduce the axon insulation function, which is a pivotal player in the high-efficient conduction of neural electrical impulses, the achievement of new skills, and the maintenance of cognition [45]. Studies of patients with Alzheimer’s disease have found that the iron concentration in deep gray matter and neocortex increases, and changes in temporal lobe iron levels are associated with cognitive decline over time [46]. Furthermore, our research has demonstrated that only GG patients or GG male patients showed obviously worse cognitive performance than GC and CC patients. Although this finding needs to be further confirmed to know whether the presence of the GG genotype may be a risk factor for more serious impairment of cognition, our results are consistent with the haplotype findings that the risk haplotype G-G in transferrin gene was significantly more frequent in schizophrenia patients than that in controls [34]. Therefore, GG variants in the transferrin gene may be involved in iron metabolism and myelination in the brain, which in turn increases the risks of detrimental cognition.

By exploring the effects of genotype on psychiatric symptoms, this study observed significant differences in the severity of negative symptoms among patients or SZ males with different rs3811655 genotypes. Furthermore, post hoc analyses showed that negative symptoms among cases carrying at least one G-allele are significantly different from that of CC patients. In particular, GG male patients showed obviously worse negative symptoms than CC ones, with an almost large effect size. It inferred that rs3811655 G carriers might exhibit more signs of avolition apathy and expressive deficit, which needs to be proved in future studies.

Interestingly, our findings elucidated that the transferrin rs3811655 polymorphism, cognition, and taking of daily antipsychotics do alone contribute minor but significant amounts to the psychopathology of schizophrenic symptoms, and rs3811655 polymorphism in particular was shown to predict the negative or total psychiatric symptoms in individuals with chronic schizophrenia. Our findings are in accordance with some current clinical data that suggest that negative symptoms may be more closely related to clinical characteristics than positive symptoms in schizophrenia [25,47], and also are affected by adverse effects of pharmacological treatment or environmental factors [48]. In addition, Kim et al. reported that patients with iron deficiency showed a higher PANSS negative symptom score, while patients with severe negative symptoms had lower serum ferritin levels but normal transferrin saturation [49]. A rodent model of inactivation of ferroportin in dopamine neurons found that the loss of transferrin receptor 1 instead of ferroportin leads to nerve iron deficiency, degeneration of dopaminergic neurons, impaired mitochondrial accumulation, and oxidative stress response, indicating neuro-degeneration of dopaminergic neurons in mice [50]. Moreover, iron plays a pivotal role in the synthesis of dopamine and its receptors. For example, iron acts as a co-factor of tyrosine hydroxylase, which is the rate-limiting enzyme for dopamine synthesis [51], whereas the D2 receptor is an iron-incorporating protein, and iron deficiency further contributes to the hypo-functionality of D2 receptors [52]. Generally, the change in the expression of the transferrin gene mediated by the rs3811655 polymorphism may lead to the dysregulation of brain iron, thus resulting in abnormal dopaminergic transmission and further facilitating the psychiatric symptoms of schizophrenia. These findings illustrated that rs3811655 polymorphism might be a potential endophenotype linked to schizophrenia and may provide clinically useful and genetically advised risk prediction for the psychiatric symptoms in cases with chronic psychosis.

Several limitations should be acclaimed in this study. First, although the exploratory analysis was conducted to examine the effects of genotype on hemoglobin in schizophrenia patients, we did not measure plasma transferrin levels or other iron-related proteins. Hence, we were unable to assess the effects of the rs3811655 polymorphism on the iron status and its correlation with phenotypes of schizophrenia. Future studies will need to further address this question. Second, it is worth noting that the patient and control samples did not match in terms of some demographic characteristics, although they were adjusted as covariates in statistical analysis, which may still cause bias in statistical analysis. In particular, the gender composition ratio of the two groups was quite different, despite our study findings affording some evidence for the impacts of transferrin gene polymorphisms on cognition in the SZ males and HC females, and a larger sample matched for gender would be more convincing. Third, TF and HFE are two genes previously claimed to affect serum transferrin levels and brain function [53,54]. In particular, the HFE H63D polymorphism was considered to alter the matter development of brain pathways that support various cognitive abilities [44]. There is a possibility that our patients may possess some of these genetic variations and have a confounding effect on the current results. Future research is required to explore more iron-related polymorphisms associated with schizophrenia. Fourth, the current study did not include first-episode schizophrenia patients with available resources, and relevant evidence should be accumulated in the early stages of the disease because antipsychotic drugs and long-term hospitalization may also affect negative symptoms and cognitive ability. Fifth, although we have provided some evidence of the homozygous GG genotype correlating with degraded cognition in schizophrenic patients, it may still be difficult to elucidate whether the effects of genotype on cognition are biased by premorbid IQ. Prospective longitudinal study designs should then be taken into account in subsequent studies. Sixth, although the RBANS could make it easy to conduct a quick comprehensive cognitive test, it is hard to predict cognitive functional outcomes in real-world settings. Therefore, in order to generalize experiment results to behaviors outside of the lab, neuropsychological assessment with high ecological validity should be used in future studies.

In summary, our study showed the same association of the polymorphism in the transferrin gene with cognitive performance in both schizophrenia patients and community controls, which may suggest that the relation is quantitative rather than qualitative: rs3811655 would not associate with the development of schizophrenia but causes more severe cognitive impairment in SZ patients. Additionally, the rs3811655 polymorphism seems to be a significant predictor of psychiatric symptoms in combination with cognition. These findings may help to understand the patterns of cognitive performance associated with transferrin gene polymorphism in patients and controls, as well as inform the potential clinical use of rs3811655 to predict pathological symptoms in patients with chronic schizophrenia.

## Figures and Tables

**Table 1:** Genotypic effects on cognitive functions between schizophrenia patients and HCs.

	Schizophrenia Patients			Healthy Controls			F_Genotypic Effects_ (*p*)	F_Case vs. Controls_ (*p*)	F_Intreaction Effects_ (*p*)
RBANS Scores	C/C (*n* = 350)	G/C (*n* = 186)	G/G (*n* = 28)	C/C (*n* = 256)	G/C (*n* = 148)	G/G (*n* = 18)			
Immediate memory	58.6 ± 16.2	59.2 ± 16.5	54.8 ± 13.9	74.0 ± 16.4	75.7 ± 17.8	70.1 ± 13.2	1.86 (0.16)	46.78 (<0.001)	0.46 (0.63)
Visuospatial	77.6 ± 18.8	77.7 ± 18.2	73.1 ± 20.8	78.4 ± 14.1	79.4 ± 15.2	76.4 ± 15.0	0.90 (0.41)	0.11 (0.74)	0.37 (0.69)
Language	81.7 ± 15.8	82.1 ± 14.4	76.8 ± 15.0	93.3 ± 12.4	94.5 ± 13.7	89.7 ± 10.6	2.41 (0.09)	50.03 (<0.001)	0.38 (0.69)
Attention	71.4 ± 17.5	70.3 ± 17.4	61.4 ± 14.7	85.8 ± 18.8	87.3 ± 19.3	82.9 ± 21.5	2.57 (0.08)	59.07 (<0.001)	1.85 (0.16)
Delayed memory	67.2 ± 19.0	65.6 ± 19.8	63.5 ± 19.1	85.0 ± 14.6	87.9 ± 14.4	81.4 ± 15.8	1.09 (0.34)	74.01 (<0.001)	2.37 (0.09)
Total index	64.7 ± 15.1	64.5 ± 14.9	60.4 ± 12.7	78.4 ± 13.7	80.7 ± 15.0	73.3 ± 14.4	3.12 (<0.05)	57.34 (<0.001)	1.97 (0.14)

**Table 2:** Clinical characteristics of patients in the rs3811655 genotype groups.

	Genotype			F/χ^2^ (*p*)
	C/C (*n* = 350)	G/C (*n* = 186)	G/G (*n* = 28)	
Age of onset (years)	23.1 ± 4.8	23.3 ± 4.8	24.4 ± 4.5	0.31 (0.73)
Duration of illness (years)	24.6 ± 8.8	24.2 ± 8.8	26.5 ± 7.6	0.23 (0.80)
Daily antipsychotic dose (mg/day) (chlorpromazine equivalent)	437.5 ± 389.7	426.8 ± 287.7	463.3 ± 443.0	0.17 (0.84)
HGB (g/dl)	141.4 ± 21.6	139.1 ± 12.0	140.6 ± 13.8	0.06 (0.94)
PANSS scores				
P subscore	11.1 ± 4.6	11.4 ± 5.0	11.0 ± 4.8	0.42 (0.66)
N subscore	21.7 ± 7.2	23.0 ± 7.3	25.1 ± 7.4	4.27 (<0.05)
G subscore	24.6 ± 5.2	25.2 ± 6.0	25.0 ± 4.9	0.81 (0.45)
Total score	57.3 ± 13.0	59.7 ± 14.8	61.1 ± 11.5	2.27 (0.10)

HGB, hemoglobin concentration.

**Table 3:** Stepwise multiple regression analysis for related factors predicting psychiatric symptoms in patients with schizophrenia.

Predictors	Stepwise Regression				Correlations	
	B (95%CI)	SE B	β	*p*	Partial *	Part ^†^
Dependent Variable: PANSS-N						
Constant	34.18 (28.08 to 40.28)	3.11		0.00		
Language	−0.11 (−0.15 to −0.06)	0.02	−0.23	0.00	−0.21	−0.18
Delayed memory	−0.06 (−0.10 to −0.02)	0.02	−0.15	0.00	−0.12	−0.11
rs3811655 genotype	0.73 (0.17 to 1.29)	0.29	0.09	0.01	0.11	0.09
Dependent Variable: PANSS-T						
Constant	54.07 (42.09 to 66.04)	6.10		0.00		
Daily antipsychotic dose	0.00 (0.00 to 0.01)	0.00	0.10	0.01	0.10	0.09
Language	−0.13 (−0.21 to −0.04)	0.04	−0.14	0.00	−0.13	−0.11
Delayed memory	−0.17 (−0.24 to −0.09)	0.04	−0.24	0.00	−0.18	−0.17
rs3811655 genotype	1.08 (−0.02 to 2.19)	0.56	0.07	0.06	0.08	0.07

β = standardized regression coefficients; B = unstandardized regression coefficients; SE = standard error. * Shared contributions of the predictors. **^†^** Unique contributions of the predictors.

## Data Availability

Data are available on reasonable request.

## Supplementary Materials

The following supporting information can be downloaded at: https://www.mdpi.com/article/10.3390/jcm11216414/s1, Table S1: Demographic and clinical data of schizophrenia patients and healthy controls; Table S2: Allele and genotype frequencies of rs3811655 polymorphism for schizophrenia and healthy controls.
