# Supplementary material for: Association of Transferrin Gene Polymorphism with Cognitive Deficits and Psychiatric Symptoms in Patients with Chronic Schizophrenia
Source: J Clin Med. 2022 Oct 29;11(21):6414. doi: 10.3390/jcm11216414 (PMC9654946; doi:10.3390/jcm11216414)
Supplement: Supplementary file 1 [file jcm-11-06414-s001.zip › Supplementary Table S1 revised.pdf]

Supplementary Table S1 Demographic and clinical data of schizophrenia patients and healthy controls

**Variable**	**Schizophrenia Patients (n=564)**	**Healthy Controls (n=422)**	**t/χ^2 (p)**
Gender (M/F)	499/65	174/248	250.65 (<0.001)
Age (years)	47.8±9.0	46.1±13.2	-2.32 (<0.05)
Education (years)	8.9±4.5	9.4±5.5	3.49 (<0.01)
Body mass index (kg/m^2)	24.8±3.9	25.3±4.0	1.82 (0.07)
Smokers (%)	396/564	151/422	133.88 (<0.001)
Age of onset (years)	23.2±4.8		
Duration of illness (years)	24.6±8.7		
Daily antipsychotic dose (mg/day) (chlorpromazine equivalent)	433.6±366.6		
PANSS scores			
P subscore	11.2±4.7		
N subscore	22.3±7.3		
G subscore	24.8±5.5		
Total score	58.3±13.6		
