# Supplementary material for: Association of Transferrin Gene Polymorphism with Cognitive Deficits and Psychiatric Symptoms in Patients with Chronic Schizophrenia
Source: J Clin Med. 2022 Oct 29;11(21):6414. doi: 10.3390/jcm11216414 (PMC9654946; doi:10.3390/jcm11216414)
Supplement: Supplementary file 1 [file jcm-11-06414-s001.zip › Supplementary Table S2 revised.pdf]

Supplementary Table S2 Allele and genotype frequencies of rs3811655 polymorphism for schizophrenia and healthy controls.

	Genotype			P(2df)	Allele		P(1df)
	**CC**	**GC**	**GG**		**C**	**G**	
Schizophrenia patients (n=564)	350(62.1%)	186(33.0%)	28(5.0%)	0.70	886(78.5%)	242(21.5%)	0.83
Healthy controls (n=422)	256(60.6%)	148(35.1%)	18(4.3%)		660(78.2)	184(21.8%)	
